# The Almoner Question

**Published:** 1921-03-26

**Authors:** 


*To the Editor of* The Hospital.

### The Almoner Question.

*To the Editor of* The Hospital.

Sir,—As a trained hospital almoner, I have read the leading article in last week's Hospital with great interest. Its meaning to the uninitiated is somewhat cryptic, but I gather that somehow, in some manner, someone has blundered. The puzzling point is that the writer of the article does not regard the blunderer as an individual, but as someone driven by a policy dictated by the Hospital Almoners' Council. As a humble member of the profession, who is not a member of the Council, may I protest against the inference? I was trained by the Council and have worked for seven years, bu the Council has never dictated, advised, or even suggested to me on the subject of my work, nor can I find that it has interfered with the work of any of my colleagues. Indeed, except for the fact that the Council notifies all almoners of vacant posts, the workers hear nothing of its activities, therefore if we do badly or well we do it as individuals.

On another point I should like to protest even more strongly. Every almoner in her training is taught tha loyalty to her hospital is of the first importance, and that her work can only be of the highest value if she co-operates with the rest of the hospital for the common good. Her work should be closely linked up with the Secretary's office, and obviously it is her duty to refer to the Secretary in all matters concerning the collection of patients' payments and question of organisation.

To come down to a small and unimportant part, the article ends with the puzzling remark that almoners are now "all the rage." The writer must surely have had grounds for the assertion, and if so it ought possibly to be cheering to those who are only conscious that they belong to a hard-working, difficult profession, which, by the very nature of its being, cannot play to the gallery, and which is still oddly unknown except by the few.

Yours faithfully,

A Hospital Almoner.

